# “Comparison of Nissen Rossetti and Floppy Nissen techniques in laparoscopic reflux surgery”

**DOI:** 10.1080/07853890.2023.2187075

**Published:** 2023-03-10

**Authors:** Cem Kaan Parsak, İlker Halvacı, Uğur Topal

**Affiliations:** Department of General Surgery, Cukurova University, Adana, Turkey

**Keywords:** laparoscopic Floppy–Nissen fundoplication, laparoscopic Nissen–Rossetti fundoplication, postoperative gastrointestinal symptoms

## Abstract

**Objective:**

The present study makes a comparative assessment of the Floppy–Nissen (FN) and Nissen–Rossetti fundoplication (NRF) procedures.

**Methods:**

Included in the study were 80 patients who presented to the General Surgery Department outpatient clinic of Balcalı Hospital of the Cukurova University Faculty of Medicine with gastroesophageal reflux between March 2010 and March 2013 All patients were operated on by the same surgeon using the laparoscopic FN or NRF techniques in a randomized controlled manner. The preoperative and postoperative reflux-specific and nonspecific gastrointestinal symptoms of the patients were compared.

**Results:**

The duration of symptoms had no effect on the level of satisfaction; regurgitation, bloating and heartburn were more common in those with a longer duration of symptoms Of the patients, 92.5% were satisfied with their resulting condition, and 92.5% were inclined toward the surgery. It was further found that there was no difference between the symptoms or satisfaction levels of the patient groups who underwent the FN procedure and those who underwent the NRF procedure, other than those related to the duration of surgery. laparoscopic NF and the NRF fundoplication treatments, aside from the duration of surgery.

**Conclusion:**

Our study revealed no significant difference between the laparoscopic NF and the NRF fundoplication treatments, aside from the duration of surgery.

**KEY MESSAGES**
The Nissen–Rossetti technique can be used safely based on the similarity of its outcomes with those of the classical Nissen technique.
Despite the documented success of laparoscopic anti-reflux surgery, the absence of studies comparing surgery and medical treatments prevents these discussions from being concluded.
Comparison of Nissen Rossetti and Floppy Nissen Techniques in Laparoscopic Reflux Surgery

## Introduction

Gastroesophageal reflux (GER) refers to the effortless, spontaneous reflux of gastric contents into the esophagus, and accounts for approximately 75% of all esophageal pathologies. It is physiologically common, especially in the postprandial period [1], and when this reflux exceeds the normal physiological limit, esophageal and extraesophageal symptoms occur. Patients may present with such typical symptoms as heartburn, chest pain, regurgitation and dysphagia, as well as such atypical symptoms as cough, hoarseness, sinusitis, pharyngitis, laryngitis and dental erosion. The easiest approach to the identification of the disease is based on symptoms, although the symptoms considered to be indicative of GERD, such as heartburn and acid regurgitation, are quite common in the general population.

The prevalence of the endoscopic detection of esophagitis in symptomatic patients is 20% – approximately 100 times higher than in the normal population [2]. The most concerning complication is Barrett’s esophagus, Barrett’s esophagus among individuals with gastro-oesophageal reflux varied according to different geographical regions ranging from 3% to 14% for histologically confirmed Barrett’s esophagus with a pooled prevalence of 7.2% (95% CI 5.4%–9.3%) Estimates of the annual cancer incidence in patients with Barrett’s esophagus have ranged from 0.1 to 0,4 percent. Although the risk of developing esophageal cancer is increased at least 30-fold above that of the general population the absolute risk of developing cancer for an individual patient with nondysplastic Barrett’s esophagus is low [3,4].

In recent years, developments in both medicine and surgery have led to an increase in discussions of the optimum treatment approach, especially between gastroenterologists and surgeons, and today the leading treatment method is considered to be PPI [5,6]. Indications for anti-reflux surgery should be based on the identification of the disease from objective values determined from appropriate tests and the presence of symptoms and should lead to the administration of an appropriate and effective medical treatment prior to surgery.

When we examine the randomized studies in the literature on the division versus non-division of short gastric vessels during Nissen fundoplication. Kinsey–Trotman in their study laparoscopic Nissen fundoplication has durable efficacy for heartburn symptom control at up to 20 years follow-up, division of short-gastric vessels failed to confer any reduction in side effects, and was associated with persistent epigastric bloat symptoms at late follow-up [7]. Similarly, Kosek et al. found that routine division of short gastric vessels during Nissen fundoplication did not provide either a functional or clinical advantage in short or long-term follow-up [8].

The present study makes a comparative assessment of the postoperative period (duration of surgery, complication development, hospital stay) and short-term outcomes (subjective assessment of, for example, the reduction of symptoms and improvement in quality of life, as well as an endoscopic demonstration of whether or not pathological acid reflux has been resolved) of the Floppy–Nissen and Nissen–Rossetti fundoplication procedures from among the laparoscopic fundoplication alternative performed for gastroesophageal reflux disease (GERD).

## Patients/materials and methods

After approval for the study was granted by the Ethics Committee of the Çukurova University Faculty of Medicine, 126 patients who presented to the General Surgery Department outpatient clinic of Balcalı Hospital of the Cukurova University Faculty of Medicine, with gastroesophageal reflux between March 2010 and March 2013 were evaluated. Excluded from the study were 16 patients with Nonerosive Reflux Disease (NERD), detected by endoscopy, and with reflux proven by a pH meter; 24 patients with endoscopic esophagitis, but with no hiatal hernia; and six patients with esophageal motility disorders detected by the manometer. The 80 study patients that remained, all of whom had endoscopic hiatal hernias and various degrees of esophagitis, were operated on sequentially using one of two procedures – a laparoscopic Floppy–Nissen procedure (group 1) or a laparoscopic Nissen–Rossetti procedure (Group 2) – for which the selection was made using a simple randomization method Randomization was done sequentially, regardless of the patients’ age, gender, education level, duration of preoperative symptoms and preoperative esophagitis degree. Floppy Nissen was applied to one patient and Rosetti Nissen to the other patient, in sequential order. Data collection and analysis were done in a blinded manner. All procedures were performed by the same surgeon (Cem Kaan Parsak MD).

At the outset of the study, the patients’ age, sex, educational level, endoscopic esophagitis grade, duration of preoperative symptoms, duration of preoperative medical treatment, year of surgery, duration of surgery, duration of postoperative follow-up, presence/absence of postoperative complications, and presence of reoperation were all recorded.

Endoscopic assessment of esophagitis were performed in accordance with the Los Angeles classification [9].

In the postoperative follow-up period, the patients were administered a satisfaction questionnaire inquiring about the presence of dysphagia, and if so, what kind of food was difficult to swallow. The evaluation was made on a scale of 0–4 (0 = no swallowing difficulties, 1 = difficulty swallowing solid food, 2 = difficulty swallowing soft food, 3 = difficulty swallowing liquids, and 4 = difficulty swallowing all).

The patients were then administered a further questionnaire to determine the levels of heartburn, bloating, frequent belching, diarrhea, abdominal pain, vomiting and inability to belch, with evaluations, again made on a scale of 0–4 (0 = no symptom, 1 = mild [noticeable but not bothersome every day], 2 = moderate [noticeable and bothersome every day], 3 = often [affecting daily life], and 4 = very often [limiting daily life]).

The patients were also administered a preoperative gastroesophageal reflux disease – health-related quality of life (GERD-HRQL) questionnaire (Appendix 1) and a gastroesophageal reflux symptoms checklist during a follow-up visit 2 months after the operation (Appendix 2). Endoscopic evaluations were performed blindly by a gastroenterology specialist at the 2nd and 12th months postoperatively. and the level of postoperative satisfaction between the two groups was compared, evaluated on a rating scale of 1–4 (1 = Very satisfied, 2 = Satisfied, 3 = Neutral [neither satisfied nor dissatisfied], and 4 = Dissatisfied). A 1-year follow-up was performed and 80 patients who participated in the study completed a one-year follow-up

Written informed consent was obtained from all participants and all study procedures were conducted according to the Declaration of Helsinki.

Statistical analyses were performed using SPSS for Windows (Version 16.0. Chicago, SPSS Inc.), and included a Wilcoxon test, a Kruskal–Wallis analysis of variance, Spearman’s correlation coefficient and a Mann–Whitney *U* test. The critical statistical significance level was set at *p* = .05 for all analyses.

### Surgical technique

All patients were operated on by the same surgeon using one of two different procedures. The following surgical steps were followed for the laparoscopic Nissen fundoplication:

Exposure of the gastrohepatic ligament; dissection of the hiatus; exposure of the gastrosplenic ligament and dissection of the fundus; closure of the crura; fundoplication.

The laparoscopic Nissen–Rossetti fundoplication, in turn, included fundoplication without exposure of the gastrosplenic ligament. The abdomen was accessed using 5-mm trocars, as previously described. After the crura were adequately identified, an opening was created posterior to the esophagus, allowing an easy pass for the fundus.

For the Floppy–Nissen procedure, the mobilization of the fundus was achieved *via* a ligature, starting at the level of an imaginary line assumed to transverse the stomach through the lower end of the spleen toward the crural. A 39-F bougie was used routinely in all patients. We recommend the surgical procedure to be standard, to facilitate dissection, and to push the bougie after the fundoplication is completed to understand whether there is a narrowing or not The crural defect was repaired with two to three non-absorbable sutures. A 2*4-cm prolen graft was fixed on the crus with a titanium ProTack™ (Covidien, U.) tacker. The fundus was passed through a window opened posterior to the esophagus, and the 360° fundoplication was completed. In this position, three non-absorbable sutures were made between the two opposing stomach walls, the first of which passed through the esophagus. Patients started to take liquids on a postoperative day 1 and were discharged with a recommendation to ingest only liquids and soft foods for 3–4 weeks after discharge.

## Results

Involved in the study were 80 patients who were assigned to two groups in a randomized controlled manner, with 40 undergoing laparoscopic Floppy–Nissen fundoplication, and 40 undergoing laparoscopic Nissen–Rossetti fundoplication. The mean age of the patients was 40.46 ± 10.396 years, and 42.5% percent were female and 57.5% were male. Of the total, 48.8% had completed higher education, while 51.2% were high school graduates or below. There was no significant difference in the age, sex or educational levels of the two groups (Table 1).

**Table 1.** Demographic characteristics.

Variables	Group 1 (Floppy–Nissen)	Group 2 (Nissen–Rossetti)	*p*
Mean age (mean ± std)	41.00 ± 10.32	39.93 ± 10.56	.647
Sex *n* (%)			.179
Female	20 (50)	14 (35)	
Male	20 (50)	26 (65)	
Educational level *n* (%)			
Primary school	5 (12.5)	4 (10)	.877
High school	15 (37.5)	17 (42.5)	
Higher education	20 (50)	19 (47.5)	

No significant difference was noted in the duration of preoperative symptoms or the duration of preoperative medical treatment between the two groups (*p* = .376; *p* = .383). Every patient underwent an endoscopic examination in the preoperative period, revealing grade A esophagitis as the most common condition in both Group 1 and Group 2 (65% vs. 60%), with no statistically significant difference between the two (*p* = .703) (Table 2).

**Table 2.** Preoperative clinical characteristics.

Variables	Group 1 (Floppy–Nissen)	Group 2 (Nissen–Rossetti)	*p*
Duration of preoperative symptoms (months), median (Min–Max)	21.0 (6–144)	24.0 (6–360)	.376
Duration of preoperative medical treatment (months), median (Min–Max)	12.0 (6–144)	15.0 (3–72)	.383
Preoperative esophagitis grade *n* (%)			.733
A	26 (65)	24 (60)	
B	10 (25)	12 (30)	
C	2 (5)	3 (7.5)	
D	2 (5)	1 (2.5)	

The duration of surgery in both groups was calculated as the time from the minute of anesthesia induction to the time of spontaneous breathing. A comparison of the two groups revealed a statistically significantly shorter operation duration in the Nissen–Rossetti group (*p* = .008). The duration of the postoperative hospital stay was the same for both surgical procedures, with a mean hospital stay of 1.39 d. Complications developed in a total of five patients, one of which required an operation. In the laparoscopic Floppy–Nissen group, three patients developed bleeding and one patient underwent surgery for an infected hematoma in the postoperative period. In the laparoscopic Nissen–Rossetti group, bleeding developed in one patient and perioperative pneumothorax in another patient, the latter of whom underwent perioperative chest tube insertion. An assessment of the complications based on the Clavien–Dindo Classification revealed two patients in Group 1 with grade I and one patient with grade IIIb, while Group 2 contained one patient with grade II and another with grade IIIa. There was no significant difference in the complications encountered between the two groups (*p* = .646). The postoperative follow-up period ranged from 12–24 months in both patient groups (Table 3).

**Table 3.** Intraoperative and postoperative clinical characteristics.

Variables	Group 1 (Floppy–Nissen)	Group 2 (Nissen–Rossetti)	*p*
Duration of operation, mean (Min–Max)	97.25 (60–180)	83.87 (45–150)	.008
Duration of postoperative stay (days), median (Min–Max)	1 (1–3)	1 (1–2)	.569
Postoperative complications, *n* (%)	3 (7.5)	2 (5)	.646
Reoperation, *n* (%)	1 (2.5)	0 (0)	.314
Duration of postoperative follow-up (days), median (Min–Max)	12 (12–24)	12 (12–24)	.143

Furthermore, three patients in the laparoscopic Floppy–Nissen group and five patients in the Nissen–Rossetti group developed difficulty in swallowing solid food (*p* = .459), the rate of postoperative bloating was 35% (*p* = .935), the rate of frequent belching was 11.3% in total (*p* = .079), the rate of inability to belch was 20% in the overall group (*p* = .267), and the rate of diarrhea was 11.3% in total (*p* = .725). Only one of the 80 patients developed postoperative vomiting, although it was not bothersome every day, and was statistically insignificant (*p* = .317). In an assessment of postoperative abdominal pain, 73.8% of the patients developed mild abdominal pain, but not occurring daily (*p* = .449). Among the 80 patients, the preoperative symptoms recurred in six patients – four in the Floppy–Nissen group and two in the Nissen–Rossetti group – and all six patients underwent medical treatment for the recurrent symptoms (*p* = .399) (Table 4).

**Table 4.** Postoperative long-term outcomes.

Variables	Group 1 (Floppy–Nissen)	Group 2 (Nissen–Rossetti)	*p*
Dysphagia *n* (%)	3 (7.5)	5 (12.5)	.459
Bloating *n* (%)	14 (35)	14 (35)	.569
Frequent belching *n* (%)	3 (7.5)	2 (5)	.646
Inability to belch *n* (%)	7 (21)	2 (5)	.267
Diarrhea	5 (12.5)	4 (10)	.725
Vomiting	1 (2.5)	0 (0)	.317
Abdominal pain *n* (%)	28 (70)	31 (77.5)	.449
Recurrence of preoperative symptoms *n* (%)	4 (10)	2 (5)	.339

The heartburn levels of the patients differed significantly between the preoperative and postoperative periods (*p* = .001), while there was no significant difference in either the preoperative or postoperative periods between the two groups (*p* = .508 for the preoperative period; *p* = .304 for the postoperative period) (Table 5).

**Table 5.** Preoperative and postoperative heartburn characteristics.

Groups	Symptoms	None	Mild	Moderate	Often	Very often	*p*
Floppy– Nissen (*n*)	Preoperative	0	0	16	23	1	.001
	Postoperative	34	5	1	0	0	
Nissen– Rossetti (*n*)	Preoperative	0	0	13	26	1	.001
	Postoperative	37	2	1	0	0	

All 80 patients underwent follow-up endoscopic examinations in our outpatient clinic at postoperative months 2 and 12. Endoscopy at postoperative month 2 revealed no esophagitis in eight patients (10%) (*p* = .951), while endoscopy at postoperative year 1 showed complete regression of esophagitis in 61 patients (76.3%) (*p* = .717) (Table 6).

**Table 6.** Comparison of endoscopy at postoperative months 2 and 12.

Month 2	Normal	Grade A	Grade B	Grade C	Grade D	*p* value
Floppy–Nissen	5	27	6	2	0	.951
Nissen–Rossetti	3	31	5	1	0	
Month 12						
Floppy–Nissen	30	6	4	0	0	.717
Nissen–Rossetti	11	7	2	0	0	

In the overall group, 43 patients were very satisfied (53.8%), 31 patients were satisfied (38.8%), five patients were neutral (6.3%) and one patient was dissatisfied (1.3%) with their resulting condition (*p* = .828) (Table 7).

**Table 7.** Distribution of postoperative satisfaction.

Groups	Very satisfied	Satisfied	Neutral	Not satisfied	*p*
Floppy–Nissen (%)	55	37	8	0	.828
Nissen–Rossetti (%)	52	40	5	3	

An examination of the relationship between preoperative symptom duration and satisfaction with the surgery revealed no negative or positive correlation between symptom duration and satisfaction level (*p* = .773) (Table 8).

**Table 8.** Comparison of duration of preoperative symptoms and levels of satisfaction.

Level of satisfaction	Duration of symptoms (mean-months)	*p* value
Very satisfied	37.12 (6–360)	.773
Satisfied	24.39 (6–108)	
Neutral	38.40 (12–72)	
Dissatisfied	36.00 (36–36)	

The overall satisfaction rate (very satisfied and satisfied) was 92.5%, and 92.5% (74/80) of the patients responded ‘yes’ when asked ‘If you developed reflux again, would you undergo this surgery?’, with no statistically significant difference between the two groups (*p* = .399). While 38 of 40 patients in the laparoscopic Floppy–Nissen group expressed that they would have the operation again, 36 of the 40 patients in the laparoscopic Nissen–Rossetti group stated that they would have the operation again.

Ppi was recommended for all patients with grade b–c esophagitis in the postoperative period, as well as 5 patients with grade esophagitis who were symptomatic.

## Discussion

The development and prevalence of laparoscopic anti-reflux surgery have led to a decline in morbidity, mortality and even surgical treatment recurrence rates [10,11]. Comparative studies between antireflux surgery and medical therapy demonstrated mixed results in patients with GERD. A large meta-analysis that included seven trials showed that surgical treatment of GERD is more effective than medical therapy with respect to patient-relevant outcomes in both the short and medium term. Heartburn and regurgitation were less frequent after surgical intervention. However, a considerable proportion of patients still needed antireflux medication after surgical fundoplication [12]. The Reflux trial, which included 21 hospitals, showed that after 5 years, laparoscopic fundoplication continued to provide better relief of GERD symptoms associated with improved health-related quality of life. Surgical complications were shown to be rare. A surgical policy has been shown to be more likely to be cost-effective, although initially more costly [13].

There are two major anti-reflux procedures: 360° total (Nissen) fundoplication and 270° partial (Toupet) fundoplication The Rossetti modification to the Nissen fundoplication built on Nissen’s original approach, involving the fixture of the anterior surface of the fundus to the anterior surface of the fundus by wrapping it around the esophagus after the complete mobilization of the abdominal esophagus and lesser curvature. Su et al. In their study in which they compared the efficacy and safety of laparoscopic Nissen, Toupet and Dor fundoplication in the treatment of hiatal hernia complicated with gastroesophageal reflux disease, these three laparoscopic fundoplications were found to be safe and feasible in the treatment of hiatal hernia complicated with GERD. However, laparoscopic Nissen and Dor fundoplication showed that it was better than Toupet fundoplication in reducing the number of reflux episodes, suppressing long reflux, increasing lower esophageal sphincter pressure, and reducing the incidence of postoperative dysphagia [14]. Du et al. found in their meta-analysis that Laparoscopic Nissen fundoplication and 180° laparoscopic anterior fundoplication (LAF) were equally effective in controlling reflux symptoms and achieved a comparable prevalence of patient satisfaction. balanced by the high risk of reoperation. They suggested that when surgeons chose surgical procedures for each individual with GERD, the risk of recurrence of symptoms should be balanced against the risk of dysphagia [15].

Randomized controlled studies have shown the Rossetti modification to be at least as successful as the classical Nissen fundoplication [16–18], or even more so [19,20]. Although the anterior wall technique seems to be an easier operation, choosing the right aspect of the anterior wall of the gastric fundus to be used in the fundoplication can be difficult, as an incorrect choice can lead to such typical laparoscopic complications as a ‘bilobed’ stomach. Other complications include a very tight valve formation, twisted fundoplication and gastric valve formation.

Clinical observations have shown that new gastrointestinal symptoms can occur and existing symptoms can continue in the postoperative period. Negre identified bothersome and unbearable gastrointestinal symptoms in 26% and 10%, respectively, of the patients in their study, while Swanstrom reported a rate of 96% [21,22]. The most likely reason for the significantly different rates reported in the literature is the assessment of groups that were not operated on by the same surgeon, as these were generally multicenter studies. Furthermore, the postoperative assessments were made by different observers, and so differences in techniques would be inevitable. The main difference in the present study is in its evaluation of patients who were operated on by the same surgeon using two different techniques, and the assessment of all patients by a single observer.

Bloating and dysphagia are the most common postoperative symptoms discussed in the literature [23–25]. There have been several studies to date identifying bloating and gas among the preoperative symptoms with the potential to occur preoperatively, and that continue to a great extent postoperatively, with a reported rate of 20–67% [21–27]. In the present study, 35% of the patients reported varying degrees of bloating, with an equal number of patients in both patient groups. All 14 patients with bloating in the laparoscopic Floppy–Nissen group described the frequency of symptoms as low, that is, mild enough not to affect daily life, while 13 patients in the laparoscopic Nissen–Rossetti group described the frequency as low and one patient as a medium, that is, at a level that affects daily life. It may be thought that bloating and gas are not symptoms specific to gastroesophageal reflux [28,29]. Another problem is dysphagia, which can be divided into its postoperative early and late forms. Postoperative dysphagia may occur for several reasons, among which are unknown motility disorders such as achalasia, peptic stricture, retroperitoneal hematoma, tight fundoplication and denervation of the lower esophagus as a result of the operation. Studies have shown that personal characteristics can also be effective, and postoperative dysphagia has been reported to be more common in patients with NERD [29–34].

Very different rates of dysphagia have been reported in the literature, with Beldi and Glattti reporting a dysphagia rate of 25% and Frantzides et al. a rate of 34% [35,36]. A comparison of the findings of the present study alongside those in the literature would suggest that dysphagia is mostly caused by solid foods. In the present study, the rate of dysphagia was 10%, and consistent with the literature, was mostly linked to solid food, with three patients in the laparoscopic Floppy–Nissen group and five patients in the laparoscopic Nissen–Rossetti group complaining of difficulty in swallowing solid food. Floppy–Nissen group and four in the laparoscopic Nissen–Rossetti group. Frequent belching is another common symptom in reflux patients. In the postoperative period, patients generally complain of being unable to belch, although frequent postoperative belching may indicate a loose fundoplication. The late appearance of frequent postoperative belching may suggest a shift either in the graft or in the fundoplication, and so it may therefore serve as an important symptom at follow-up. In the present study, the rate of frequent postoperative belching was 11.3%.

The mechanism of an inability to belch, as another common symptom after anti-reflux surgery, can be explained as follows: The reflex required to belch begins with the stimulation of tension receptors in the fundus. The dissection of short gastric vessels during the operation may lead to the dissection of the afferent nerves required for this reflex, resulting in the loss of reflex, leading potentially to an inability to belch in the postoperative period. A previous study found the rate of inability to belch to be 22%, while our study established a rate of 20%, which is consistent.

Regurgitation and heartburn are the two main symptoms of GERD. It should not be assumed that patients who express ongoing heartburn are experiencing a recurrence of reflux, as continuing symptoms may be attributable to the previous esophageal irritation, and it may take several months for this to fully resolve. Many studies have reported that these symptoms largely resolve within three months [36,37]. Our study identified regurgitation to various degrees in each patient in the preoperative period, while this rate decreased to 11.3% in the postoperative period and mostly occurred only once a week with little bother to the individual (*p* = .001). Heartburn and regurgitation were detected more often in the postoperative period in patients with a longer duration of preoperative symptoms.

In the present study, the rate of taking medication due to newly developed symptoms in the postoperative period was 8.8%, while a rate of 14% was reported in a study comparing postoperative 5–8 year outcomes [26] in which 79% received treatment for symptoms unrelated to reflux. It was established in the present study that the patients used mainly simethicone-group drugs for the treatment of bloating, and a small proportion took PPI irregularly, but without a physician’s recommendation and without pathology.

Despite all of these postoperative symptoms, 92.5% of the patients were satisfied with their current condition, and 92.5% responded ‘yes’ when asked ‘If you developed reflux again, would you have this surgery?’ Only 7.5% of the patients had recurrent symptoms and 8.8% were undergoing irregular medical treatment.

Laparoscopic fundoplication procedures have proven to be successful for the treatment of gastroesophageal reflux disease with low morbidity. As can be seen, gastrointestinal symptoms occur at various rates after laparoscopic surgery, and multiple theories have been put forward to explain the mechanisms behind their occurrence. There are different mechanisms behind the development of different symptoms, including vagal injury, tight fundoplication, the shift of the fundoplication into the thorax, dietary habits and air swallowing [21,38]. A previous study found postoperative symptoms to be more common when vagotomy was added to anti-reflux surgery [39], suggesting that vagal injury during laparoscopic anti-reflux surgery may lead to the development of gastrointestinal symptoms in the postoperative period. It is believed that postoperative adhesions may also be an effective factor delaying gastric and duodenal emptying, although these dyspeptic symptoms may also be related to an underlying undiagnosed disease. In such cases, the operation may not be the direct cause of the symptoms but may play a supporting role in their emergence. Nissen recommends care during surgery not to cause vagal injury [21,40].

The high patient satisfaction rate, even in the presence of gastrointestinal symptoms, is proof that the operation is effective and well-tolerated. The main determinant here, however, is the frequency and severity of symptoms. One of the factors determining satisfaction with the operation relates to patient expectations from the operation. Obviously, if patients are told that they may experience such postoperative symptoms as bloating, inability to belch, vomiting and sometimes diarrhea, and that these symptoms may occur depending on the physiology of the surgery, patient satisfaction will be increased.

The limitations of our study were that the data were obtained based on verbal statements and that some of the data were relatively subjective.

Our study concluded that the surgical treatment option can be chosen for the treatment of GERD, and that the Nissen–Rossetti technique can be used safely based on the similarity of its outcomes with those of the classical Floppy–Nissen technique, but with a shorter operation duration.

## Data Availability

The data supporting the findings of this study are available from the corresponding author, [Ugur Topal), upon reasonable request.

## Appendix 1. GERD – HRQL

**Table:**

Gastroesophageal Reflux Disease – Health-Related Quality of Life Questionnaire						
How bad is your heartburn?	0	1	2	3	4	5
Heartburn when lying down?	0	1	2	3	4	5
Heartburn when standing up?	0	1	2	3	4	5
Heartburn after meals?	0	1	2	3	4	5
Heartburn when you change your diet?	0	1	2	3	4	5
Does heartburn wake you from sleep?	0	1	2	3	4	5
Do you have difficulty swallowing?	0	1	2	3	4	5
Do you have pain when swallowing?	0	1	2	3	4	5
Do you take medication? Does this affect your life?	0	1	2	3	4	5
How satisfied are you with your present condition?	Satisfied		Neutral		Dissatisfied	
0 = No symptom 1 = Symptoms noticeable but not bothersome 2 = Symptoms noticeable and bothersome, but not every day 3 = Symptoms bothersome every day 4 = Symptoms affect daily activities 5 = Symptoms are incapacitating, affecting daily activities						

## Appendix 2. Gastroesophageal reflux symptoms checklist

**Table:**

Full Name:		Age: Sex:				
Educational Level:		Duration of operation (min):				
Duration of preoperative symptoms:		Duration of perioperative medical treatment:				
Duration of hospital stay:		Reoperation:				
Complications:		Duration of postoperative follow-up				
1. Do you have difficulty swallowing?						
0	1	2	3		4	
2. Do you experience early satiety?						
0	1	2	3		4	
3. Do you experience water brash?						
0	1	2	3		4	
4. Do you have heartburn?						
0	1	2	3		4	
5. Do you have bloating?						
0	1	2	3		4	
6. Do you experience frequent belching?						
0	1	2	3		4	
7. Do you experience frequent diarrhea?						
0	1	2	3		4	
8. Do you experience abdominal pain?						
0	1	2	3		4	
9. Do you experience vomiting?						
0	1	2	3		4	
10. Do you experience inability to belch?						
0	1	2	3		4	
Item 1 - Rating Scale						
0: no difficulty swallowing				1: difficulty swallowing solid food		
2: difficulty swallowing soft food				3: difficulty swallowing liquids		
4: difficulty swallowing all						
Item 2 - Rating Scale						
0: no early satiety				1: early satiety (feels full after 1/2 bowl)		
Items 3–10 - Rating Scale						
0: no (No symptoms)						
1: mild (Symptoms noticeable but not bothersome every day)						
2: moderate (Symptoms noticeable and bothersome every day)						
3: often (Symptoms affect daily activities)						
4: very often (Symptoms are incapacitating, affecting daily activities)						
11. How satisfied are you with your present condition?						
1: Very satisfied				2: Satisfied		
3: Neutral				4: Dissatisfied		
12. Do you experience recurrence in your symptoms leading to surgery?						
0: No				l: Yes		
13. Do you take medication for your new symptoms?						
Yes				No		
14. If you developed reflux again, would you have this surgery?						
Yes				No		
